# M2 Macrophages Activate WNT Signaling Pathway in Epithelial Cells: Relevance in Ulcerative Colitis

**DOI:** 10.1371/journal.pone.0078128

**Published:** 2013-10-22

**Authors:** Jesús Cosín-Roger, Dolores Ortiz-Masiá, Sara Calatayud, Carlos Hernández, Angeles Álvarez, Joaquin Hinojosa, Juan V. Esplugues, Maria D. Barrachina

**Affiliations:** 1 Departamento de Farmacología and CIBERehd, Facultad de Medicina, Universidad de Valencia, Valencia, Spain; 2 FISABIO, Hospital Dr. Peset, Valencia, Spain; 3 Hospital de Manises, Valencia, Spain; Massachusetts General Hospital, United States of America

## Abstract

Macrophages, which exhibit great plasticity, are important components of the inflamed tissue and constitute an essential element of regenerative responses. Epithelial Wnt signalling is involved in mechanisms of proliferation and differentiation and expression of Wnt ligands by macrophages has been reported. We aim to determine whether the macrophage phenotype determines the expression of Wnt ligands, the influence of the macrophage phenotype in epithelial activation of Wnt signalling and the relevance of this pathway in ulcerative colitis. Human monocyte-derived macrophages and U937-derived macrophages were polarized towards M1 or M2 phenotypes and the expression of *Wnt1* and *Wnt3a* was analyzed by qPCR. The effects of macrophages and the role of Wnt1 were analyzed on the expression of β-catenin, Tcf-4, c-Myc and markers of cell differentiation in a co-culture system with Caco-2 cells. Immunohistochemical staining of CD68, CD206, CD86, Wnt1, β-catenin and c-Myc were evaluated in the damaged and non-damaged mucosa of patients with UC. We also determined the mRNA expression of *Lgr5* and *c-Myc* by qPCR and protein levels of β-catenin by western blot. Results show that M2, and no M1, activated the Wnt signaling pathway in co-culture epithelial cells through Wnt1 which impaired enterocyte differentiation. A significant increase in the number of CD206+ macrophages was observed in the damaged mucosa of chronic *vs* newly diagnosed patients. CD206 immunostaining co-localized with Wnt1 in the mucosa and these cells were associated with activation of canonical Wnt signalling pathway in epithelial cells and diminution of alkaline phosphatase activity. Our results show that M2 macrophages, and not M1, activate Wnt signalling pathways and decrease enterocyte differentiation in co-cultured epithelial cells. In the mucosa of UC patients, M2 macrophages increase with chronicity and are associated with activation of epithelial Wnt signalling and diminution in enterocyte differentiation.

## Introduction

Inflammatory bowel disease (IBD) is associated with disruption of the epithelial barrier function. The control of inflammation has been for years the main focus of research, but there is growing awareness that complete repair of the epithelial layer must be achieved for long-term remission of IBD [1–3]. Regeneration of the mucosa depends on the coordinated regulation between proliferation and differentiation into epithelial cell lineages of the progenitor cells, a process that is mainly regulated by the Wnt signalling pathway [4–6]. This pathway includes a group of ligands that act as intercellular signalling molecules regulating the cellular fate along the crypt in normal gut epithelium and in response to epithelial injury. Upon binding to their receptors, canonical Wnt ligands induced inactivation of GSk3β and accumulation and nuclear translocation of β-catenin where it engages DNA bound TCF transcription factors [7–9]. Activation of the canonical Wnt pathway is observed in cells located at the base of the crypts and it functions to maintain the crypt cell population in a proliferative state while down regulates the differentiation process. 

There is strong evidence that the inflammatory microenvironment modulates intestinal wound healing [10,11]. Macrophages constitute one of the central components of inflamed tissue and are considered an essential element of regenerative responses [12,13]. Several studies have reported synthesis of Wnt ligands by macrophages [13,14].These cells exhibit great plasticity and it has been proposed the existence of two macrophage subsets which differ substantially in terms of receptor expression, effector function and cytokine production [15,16]. M1, or classically activated, macrophages are characterized by the expression of high levels of pro-inflammatory cytokines and mediate antitumor immunity and defence of the host from microorganisms. On the other hand, M2, or alternatively activated, macrophages express high levels of anti-inflammatory cytokines and scavenging molecules while exerting anti-inflammatory actions and regulating wound healing. Despite these important differences, the polarization of macrophages is not definitive, as they retain the capacity to convert into the other phenotype in accordance with the local microenvironment. Mucosal microenvironment can vary dramatically depending on the form of human IBD, Ulcerative colitis (UC) or Crohn's disease (CD), the chronicity or the stage of the disease. However, there is little knowledge of the activation pattern of macrophages in this pathology and its significance for intestinal mucosal healing. 

The purpose of this study was to analyse the involvement of the macrophage phenotype in the activation of Wnt signalling pathways in intestinal epithelial cells and to evaluate its relevance in the mucosa of UC patients. We found that, in contrast to M1 macrophages, M2 macrophages overexpressed Wnt ligands and activated Wnt signalling pathways in epithelial cells which reduced markers of differentiation. In the damaged mucosa of UC patients the number of M2 macrophages increased with chronicity and it was associated with activation of Wnt signalling pathways and diminution in enterocyte differentiation.

## Material and Methods

### Cell culture

Caco-2 cells (American Type Culture Collection, VA, USA) were cultured in MEM medium (Sigma-Aldrich) supplemented with 20% inactivated bovine foetal serum, 100 U/ml penicillin,100 μg/ml streptomycin, 2 mM L-glutamine, 100 mM sodium pyruvate and 1% of non-essential amino acids. HT29 (American Type Culture Collection, VA, USA) were cultured in McCoy’s Medium Modified (Sigma-Aldrich) supplemented with 10% inactivated bovine foetal serum, 100 U/ml penicillin, 100 μg/ml streptomycin and 2 mM L-glutamine. When appropriated, epithelial cells were treated with exogenous Wnt1 (20ng/ml, Sigma-Aldrich).

U937 human monocytes (European Collection of Cell Culture, Salisbury, UK) were cultured in RPMI medium (Sigma-Aldrich CO, St. Louis, MO) with 10% inactivated bovine foetal serum (FBS, Lonza, Basel, Switzerland), 100 U/ml penicillin and 100 μg/ml streptomycin. U937 monocytes were differentiated into macrophages by culturing them in the presence of phorbol-12-myristate-13-acetate (PMA, Sigma-Aldrich) for 48 h[17]. U937-derived macrophages were stimulated with LPS (0.1µg/ml; *E. coli* 0111:B4) and IFN-γ (20 ng/ml) or with IL-4 (20 ng/ml) in order to polarize them towards M1 or M2 phenotypes, respectively. To determine the most effective period for polarization the expression of several markers was analyzed at different time points (0, 8, 24, 48, 72, and 96 hours). 

### RNA interference and cellular transfection

U937-derived macrophages cells were transfected with a vector-targeting human Wnt1 (miWnt1, targeting sequence: 5’TGACTTGTTAAACAGACTGCGAA3’, GenBank Accession No. NM_005430) or a non-targeting control vector (mock). Lipofectamine-2000 (Invitrogen Life Technologies, Barcelona, Spain) was employed as a transfection reagent and used as previously reported [18]. Sixteen hours post-transfection macrophages were polarized into M2 macrophages as described above. The efficiency of transfection was determined by analyzing the *Wnt1* mRNA expression by qPCR.

### Co-culture

Caco-2 cells were co-cultured with U937-macrophages using Transwell^®^ inserts (Corning Incorporated, MA, USA) with a 0.4 µm porous membrane [18]. U937-derived macrophages were seeded on the inserts and differentiated properly. Afterwards the inserts were placed on top of Caco-2 cells and were maintained in co-culture for 24 hours. When appropriated, Caco-2 cells were treated with the inhibitor of the Wnt-pathway, XAV939 (1 µM, Sigma-Aldrich) or with Wnt1 (20ng/ml, Sigma-Aldrich).

### Isolation of mononuclear cells

Human peripheral blood mononuclear cells were isolated from healthy donors and UC patients by Ficoll density gradient centrifugation at 400g during 40 minutes. Monocyte-derived macrophages (MDMs) were obtained from monocytes which were seeded in 6-well tissue culture plates and differentiated into macrophages by culturing them in X-Vivo 15 medium (Lonza, Basel, Switzerland) supplemented with 1% human serum, 100 U/ml de penicillin, 100 μg/ml streptomycin and 20 ng/ml recombinant human M-CSF (Peprotech, London, UK) at 37°C in 5% CO_2_ for 6 days. In order to obtain an M1 polarization, cells were incubated with 0.1µg/ml LPS (from *Escherichia coli* 0111:B4) plus 20 ng/ml human recombinant IFNγ for the last 24 hours. M2 polarization was obtained by treating cells with 20ng/ml of human recombinant IL-4 for the last 48 hours of the culturing period. 

### Patients

A group of chronic patients with ulcerative colitis (UC) and newly diagnosed UC patients underwent a colonoscopy or sigmoidoscopy during which multiple biopsy specimens were taken from damaged and non-damaged mucosa (Table S1). Clinical disease activity was determined by the Mayo Clinic Index and in all cases active disease was observed. Histological analysis of the damaged mucosa was performed in representative 5 µm sections of paraffin-embedded tissue stained with hematoxilin and eosine. Sections were scored histologically by personnel blinded to the clinical information according to a scale of 1 (intact mucosa and normal villous mucosal surface), 2 (dilated crypts, branching crypts), 3 (increased intercrypt distance) or 4 (near absence of crypts). Colonic surgical resections from both damaged and non-damaged mucosa were also obtained from UC patients (n=8).

### Immunohistochemical studies

Immunostaininig for CD68, CD86, CD206, β-catenin, Wnt1 and c-Myc was performed in 5 µm sections of paraffin-embedded colonic tissues (Table 1). A horse anti-mouse/rabbit biotinylated antibody (Vector Laboratories, CA, USA, 1:200) was used as a secondary antibody. The VECTASTAIN elite ABC system Kit (Vector Laboratories) was employed for signal development. All tissues were counterstained with hematoxylin and the specificity of the immunostaining was confirmed by the absence of primary or secondary antibodies. Quantitative analysis of macrophages was performed in an area of 0.3 mm^2^. The software ImageJ (National Institutes of Health, Bethesda, MD, USA) was used to quantify the intensity of β-catenin staining. Three representative crypts of each sample were evaluated and results were normalized to the area of each crypt.

**Table 1:** Specific antibodies used for both immunohistochemical studies and Western blot analysis.

**Antibody**	**Immunohistochemistry**		**Western blot**
	Antigen Retrieval	Antibody dilution	Antibody dilution
CD68 (Biolegend, Madrid, Spain)	α-chymotrypsin 37°C 20 min	1:100	
c-Myc (Santa Cruz Biotechnology, Heidelberg, Germany)	sodium citrate buffer pH=6 97°C20 min	1:100	
Wnt1 (Sigma-Aldrich, CO, St. Louis, MO)	sodium citrate buffer pH=6 97°C20 min	1:400	
CD86 (Epitomics, Burlingame, CA, U.S.A.)	α-chymotrypsin 37°C 20 min	1:200	1:500
CD206(Sigma-Aldrich)	sodium citrate buffer pH=9 97°C 20 min	1:200	1:500
β-catenin(Sigma-Aldrich)	sodium citrate buffer pH=6 97°C 20 min	1:200	1:1000
β-actin(Sigma-Aldrich)			1:10000
Nucleolin(Sigma-Aldrich)			1:2500

### Double Immunohistochemistry

After visualization of the expression of the first primary antibody (Wnt1) with DAB, sections were washed in PBS and excess of biotin and avidin were blocked with Dako Cytomation Biotin Blocking System (Dako, Barcelona, Spain). Tissues were blocked and were incubated with the second primary antibody (CD206). After incubation with the secondary antibody, the second signal was developed with Vector Purple (Vector Laboratories). The specificity of the immunostaining was confirmed by the absence of both primary antibodies.

### Protein extraction, western blot analysis and immunoprecipitation

Equal amounts of protein from U937-macrophages, Caco-2 cells or colonic tissue were loaded onto SDS/PAGE gels and analyzed by Western blot as described previously [19]. To determine nuclear β-catenin, proteins were extracted by sonication of nuclear pellets followed by centrifugation. Membranes were incubated overnight at 4°C with different primary antibodies (Table 1). Subsequently, membranes were incubated with a peroxidase-conjugated anti-mouse IgG (Thermo Scientific, Rockford, IL, U.S.A., 1:5000) or anti-rabbit IgG (Thermo Scientific, 1:10000). Following treatment with supersignal west pico chemiluminescent substrate (Thermo Scientific), protein bands were detected by a LAS-3000 (Fujifilm, Barcelona, Spain). Protein expression was quantified by means of densitometry using Image Gauge Version 4.0 software (Fujifilm). Data were normalized to β-actin or nucleolin. 

For immunoprecipitation [20], 200µg of total protein was diluted in extraction buffer and was incubated overnight with affinity-purified monoclonal antibody against Tcf-4 (Millipore Iberica, Spain, Madrid) under agitation at 4°C. After binding to protein A-Sepharose CL-4B (GE Health-care, UK) for 4h at 4°C under agitation, the immunoprecipitates were washed three times with extraction buffer. In order to denature the protein and separate it from the protein-A beads, loading buffer 2X was added and boiled at 100°C. The supernatants were analyzed by immunoblotting analysis.

### RNA extraction and PCR analysis

Total RNA was isolated from macrophages or Caco-2 cells by using the extraction kit (Illustra RNAspin Mini, GE HealthCare Life Science, Barcelona, Spain) and total RNA from colonic tissue was isolated using the Tripure Isolation reagent (Roche, Spain). cDNA was obtained with the Prime Script RT reagent Kit (Takara Biothecnology, Dalian, China). Real-time PCR was performed with the Prime Script Reagent Kit Perfect Real Time (Takara Biotechnology) in a thermo cycler LightCycler (Roche Diagnostics, Mannheim, Germany). Specific oligonucleotides were designed according to sequences shown in Table 2. Relative gene expression was quantified as previously described [17].

**Table 2:** Primer sequences of specific PCR products for each gene analyzed.

Human Gene	Sense	Antisense	Length (bp)
*iNOS*	5´-TCAGCAAGCAGCAGAATGAG-3´	5´-TCAGCAAGCAGCAGAATGAG-3´	213
*Arginase*	5´-AGGGACAGCCACGAGGAGGG-3´	5´-AGTTTCTCAAGCAGACCAGCCTTTC-3´	70
*Wnt1*	5´-CGCCCACCCGAGTACCTCCA-3´	5´-TTCATGCCGCCCCAGGCAAG-3´	110
*Wnt3a*	5´-TACTCCTCTGCAGCCTGAAGCA-3´	5´-ATGGCGTGGACAAAGGCCGAC-3´	322
*c-Myc*	5`-TTCCCCTACCCTCTCAACGAC-3`	5`-TTCTTCCTCATCTTCTTGTTCCTCC-3`	201
*Lgr5*	5`-GGCTCGGTGTGCTCCTGTCCT-3´	5´-TGCCTCAGGGAATGCAGGCC-3´	484
*β-actin*	5´-GGACTTCGAGCAAGAGATGG-3´	5´-AGCACTGTGTTGGCGTACAG-3´	67

### Alkaline phosphatase activity

Caco-2 or HT29 cells were washed with cold PBS and lysed in 150 μl of 0.5% Triton X-100, 10mM Tris-HCl [pH=8] and 150mM NaCl. Frozen non-damaged and damaged mucosa were homogenated with Ultraturrax T25 basic IKA-Werke three times during 10 seconds and centrifuged 30 minutes at 13200 rpm at 4°C. Each sample was mixed with p-nitrophenyl phosphate solution (Sigma-Aldrich). Thirty minutes later, absorbance at 405nm was measured. Protein content was quantified using Bradford Assay (Bio-Rad Laboratories, Madrid, Spain).

### Statistical analysis

Data were expressed as mean±SEM and compared by analysis of variance (one way-ANOVA) with a Newman-Keuls post hoc correction for multiple comparisons or an unpaired Student´s *t* test where appropriate. A *P* value of <0.05 was considered to be statistically significant. The clinical correlations were analyzed using Spearman’s correlation coefficient.

### Ethical Considerations

The study was approved by the Institutional Review Board of The Hospital of Manises (Valencia, Spain). Written informed consent was obtained from all patients.

## Results

### M2 macrophages induce the expression Wnt ligands

**Figure 1:**
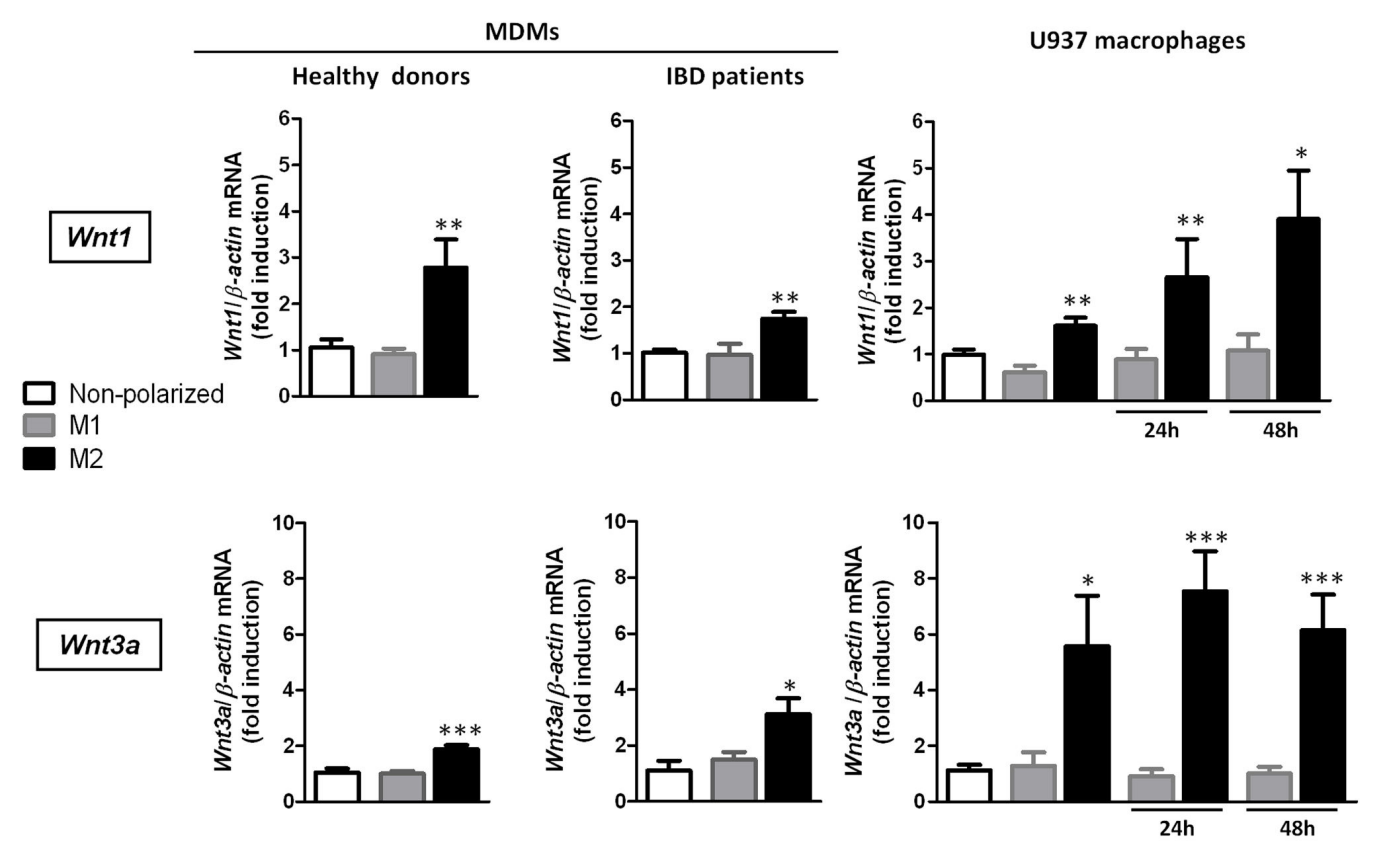
The expression of Wnt ligands is selectively induced by M2 macrophages. A) Human monocyte-derived macrophages (MDMs) from healthy donors (n=6) or IBD patients (n=6) and U937-derived macrophages (n=6) were either treated with LPS and IFN-γ and polarized towards M1 macrophages or treated with IL-4 and polarized towards M2 macrophages; some cells were treated with vehicle (non-polarized macrophages). In all graphs the first three columns refers at the moment in which polarization is obtained and the other columns refer 24h or 48h after polarization Graphs show relative mRNA expression levels of two Wnt ligands, *Wnt1* and *Wnt3a* in macrophages vs the housekeeping gene *β-actin*. Bars represent mean±SEM. *P<0.05, **P<0.01 and ***P<0.001 *vs* respective non-polarized or M1 macrophages at the same time point.

Human monocyte-derived macrophages from healthy donors and UC patients and macrophages derived from U937 cells (Figure S1) were polarized to the M1 or M2 phenotype and we analysed the expression of *Wnt1* and *Wnt3a* in these cells. In all cases, the mRNA expression of the canonical Wnt ligands, *Wnt1* and *Wnt3a*, was significantly increased in M2 cells when compared with the expression observed in both M1- and non-polarized macrophages (Figure 1).

### M2 macrophages activate the Wnt signalling pathway in epithelial cells

**Figure 2:**
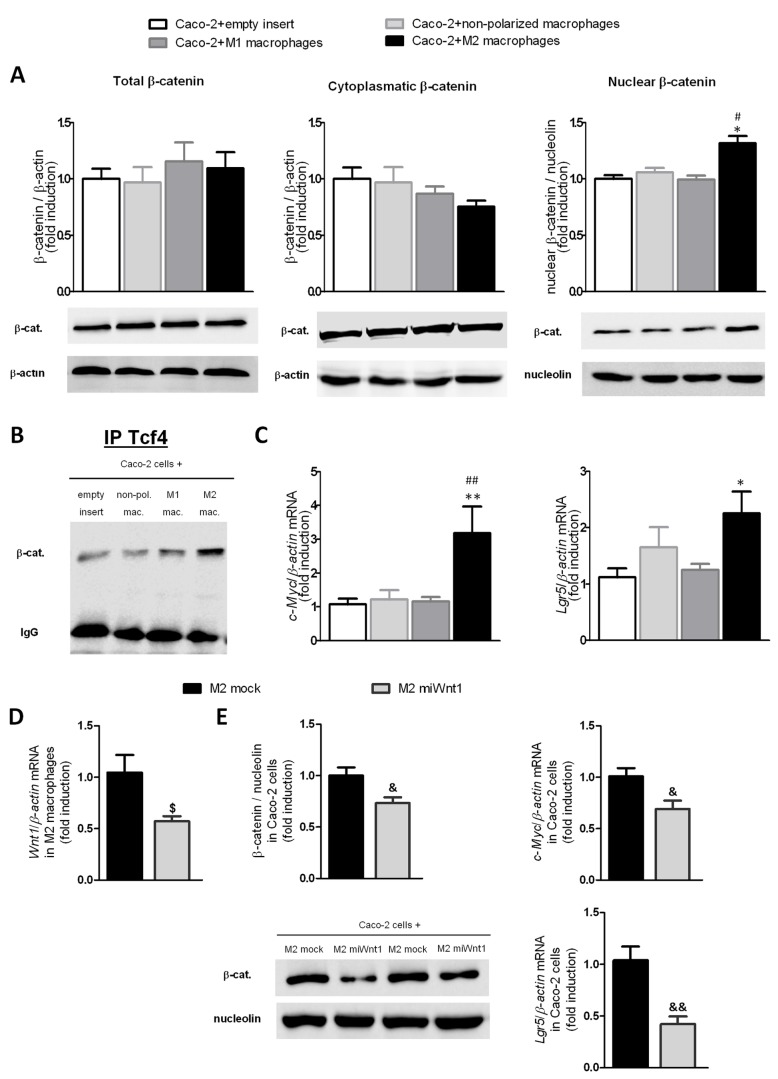
M2 macrophages activate the Wnt signalling pathway in epithelial cells. Caco-2 cells were co-cultured for 24h with M1, M2 or non-polarized macrophages derived from U937 cells or an empty insert. A) A representative western blot and graph showing protein expression of total, cytoplasmatic and nuclear β-catenin (n=4) in Caco-2 cells after co-culture. B) A representative western blot of β-catenin after immunoprecipitation of Tcf-4 of three experiments. C) Graphs show the mRNA expression levels of *c-Myc* (n=7) and *Lgr5* (n=7) in Caco-2 cells after co-culture. D) Graphs show the mRNA expression level of *Wnt1* in M2 macrophages treated with *miWnt1* or *mock*. E) Caco-2 cells were co-cultured with transfected M2 macrophages. Graphs show quantification of protein expression of nuclear β-catenin by western blot (n=5) and mRNA expression of *Lgr5* (n=5) and *c-Myc* (n=5). Two representative western blots showing nuclear β-catenin in Caco-2 cells. Bars represent mean±SEM. *P<0.05 and **P<0.01 *vs* Caco-2 cells co-cultured with an empty insert. ^#^ *P*<0.05 and ^##^ *P*<0.01 *vs* Caco-2 cells co-cultured with M1 macrophages or non-polarized macrophages. ^$^ *P*<0.05 vs M2 macrophages transfected with mock and ^&^ *P*<0.05 and ^&&^ *P*<0.01 vs Caco-2 cells co-cultured with M2 macrophages transfected with mock.

To determine the influence of the Wnt ligands expression by macrophages on epithelial cells, we established a co-culture system in which macrophages and epithelial cells were in close proximity and shared the culture medium. As shown in Figure 2A, M2 macrophages induced a significant increase in nuclear accumulation of β-catenin in Caco-2 cells compared with that induced by M1 or non-polarized macrophages while non-significant differences were observed in total or cytoplasmic β-catenin in Caco-2 cells co-cultured with different macrophage phenotypes. Analysis of β-catenin expression in Tcf-4 immunoprecipitates revealed an increase of the amount of β-catenin attached to Tcf-4 in those Caco-2 cells that had been co-cultured with M2 macrophages when compared with the other experimental groups (Figure 2B). Furthermore, results also show an increase in the mRNA levels of *Lgr5* and *c-Myc*, two target genes of the Wnt route, in Caco-2 cells that had been co-cultured with M2 macrophages while no significant induction of these genes was observed in neither cells co-cultured with non-polarized nor M1 macrophages (Figure 2C).

The role of Wnt1 released from M2 macrophages on the epithelial activation of Wnt pathway was determined using a miRNA approach to selectively knockdown Wnt1 by transient transfection in M2 cells (Figure 2D). Results showed a diminution of nuclear β-catenin expression in those Caco-2 cells that had been co-cultured with M2 macrophages transfected with *miWnt1* compared with cells transfected with mock (Figure 2E). Furthermore, the mRNA expression of *Lgr5* and *c-Myc* was also significantly diminished when epithelial cells were co-cultured with M2 macrophages transfected with miWnt1 compared with cells transfected with mock.

### M2 macrophages impair enterocyte differentiation through activation of the Wnt signalling pathway

**Figure 3:**
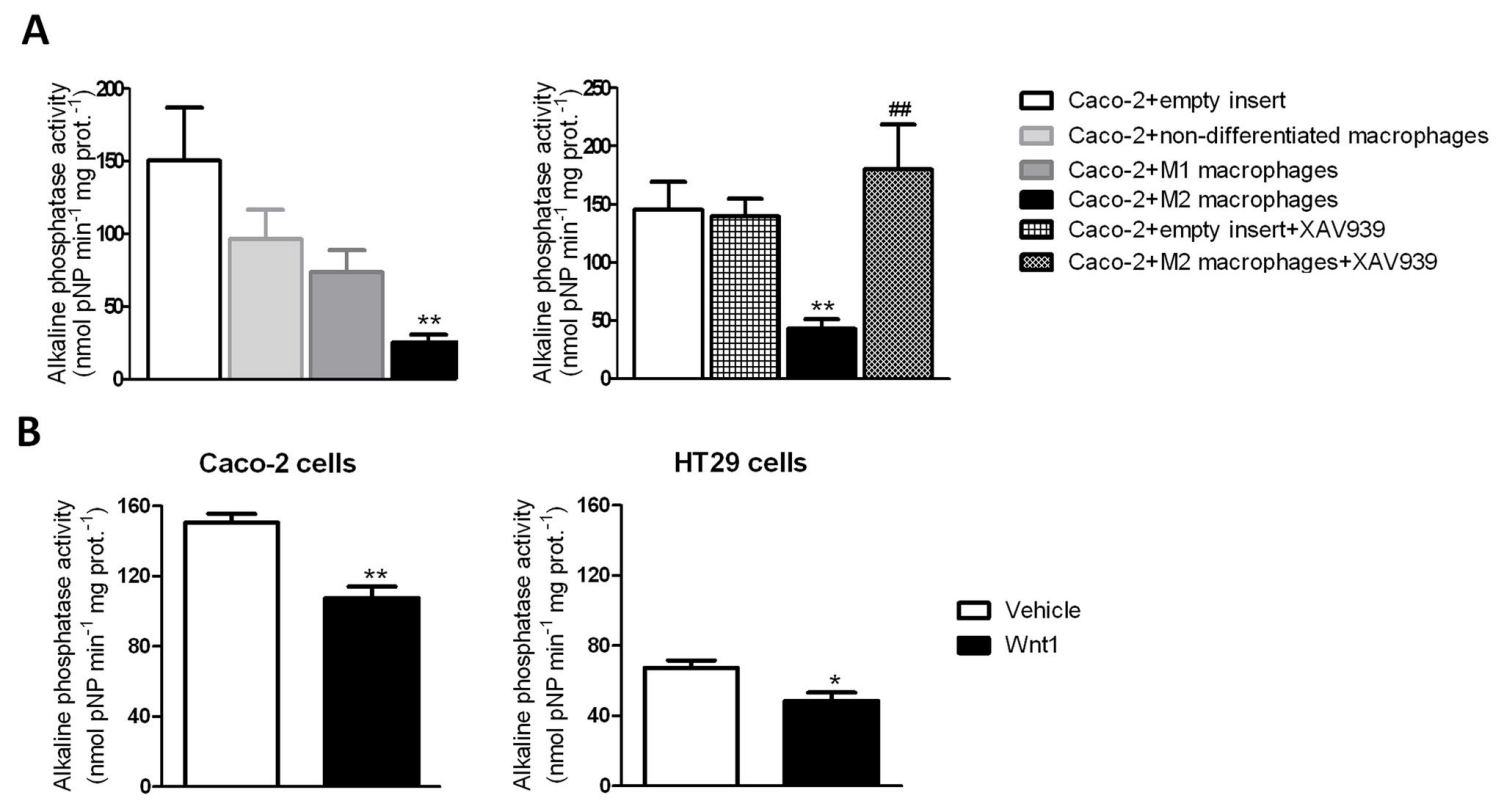
M2 macrophages decrease alkaline phosphatase activity through Wnt signalling pathways. A) Graphs show alkaline phosphatase activity in Caco-2 cells co-cultured with macrophages or with an empty insert (n=5). Some cells were treated with XAV939, 1µM (n=4). Bars represent mean±SEM. **P<0.01 *vs* Caco-2 cells co-cultured with an empty insert and ^##^ *P*<0.01 *vs* Caco-2 cells co-cultured with M2 macrophages. B) Graphs show alkaline phosphatase activity in Caco-2 cells and HT29 cells 24h after treatment with Wnt1 20ng/ml. Bars represent mean±SEM. *P<0.05 and ** *P*<0.01 *vs* cells treated with vehicle.

We next evaluated the influence of macrophages on a well-established marker of cell differentiation, alkaline phosphatase (AP) activity and the involvement of the Wnt pathway. Co-culture with any macrophage phenotype induced a reduction of alkaline phosphatase activity in Caco-2 cells but only those co-cultured with M2 macrophages underwent a significant diminution (Figure 3A). This effect was abolished by treatment with XAV939 (Figure 3A), thus implicating the Wnt pathway in the diminution observed. The role of Wnt signaling in modulating the enzymatic activity was further reinforced by experiments showing that the exogenous administration of Wnt1 to epithelial cells induced a significant reduction in AP activity in both, Caco-2 and HT29 cells (Figure 3B). As a whole, these results suggest that activation of the Wnt pathway by M2 macrophages impairs enterocyte differentiation in Caco-2 cells.

### M2 macrophages are increased in the damaged mucosa of chronic UC patients

**Figure 4:**
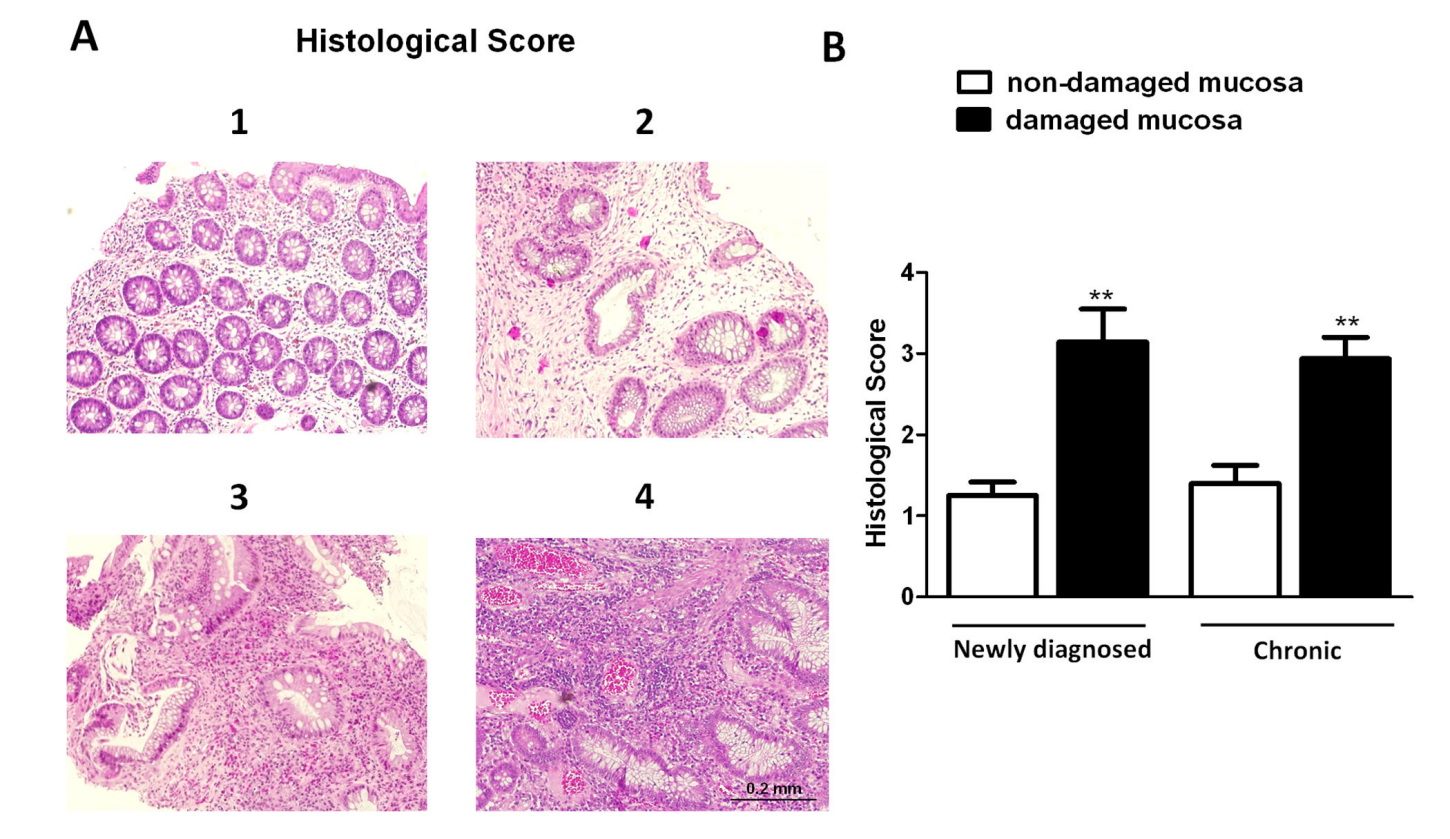
Histological score in the mucosa of patients with UC. A) Representative photographs showing histological score assigned to biopsies, magnification 10X. B) Graphs show histological score in damaged and non-damaged mucosa of newly diagnosed (n=8) and chronic UC patients (n=12). Bars represent mean±SEM. **P<0.01 *vs* respective non-damaged mucosa.

From a histological point of view, the architecture of mucosa defined as non-damaged during biopsy was preserved in both, patients at diagnosis and chronic patients. However, mucosal samples from the same patients defined as damaged during colonoscopy exhibited substantial changes in the structure of the tissue, with the presence of dilated and branching crypts and a considerable distance between crypts (Figure 4A, B). In these samples, no significant differences in the histological score were observed between patients at diagnosis and chronic patients (Figure 4B).

**Figure 5:**
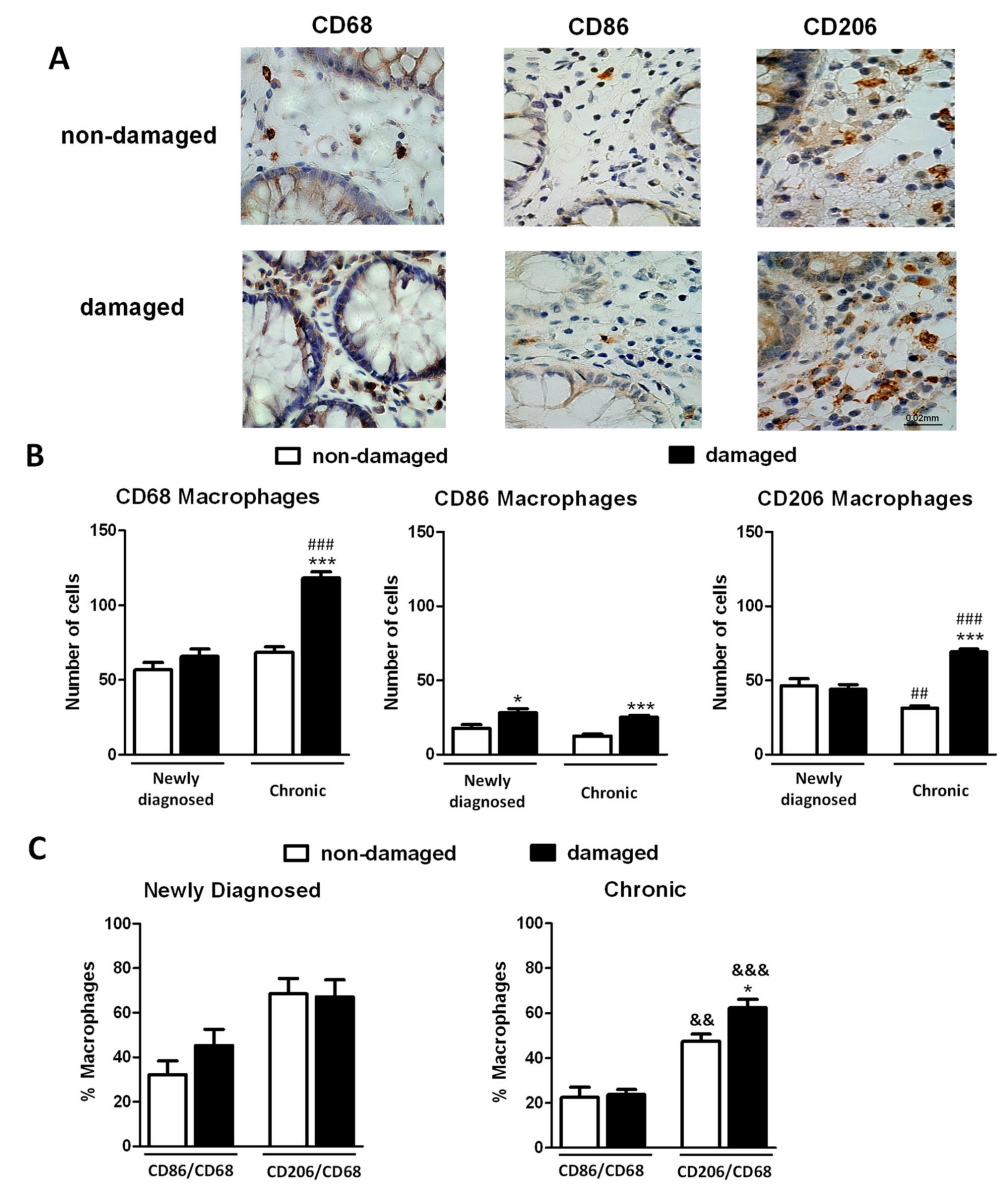
M2 macrophages increase in the mucosa of chronic UC patients. A) Representative photographs showing CD68, CD86 and CD206 immunostaining in paraffin-embedded sections of damaged and non-damaged mucosa of patients with UC, magnification 60X. B) Graphs show quantitative analysis of CD68, CD86 and CD206 positive cells performed in a total area of 0.3 mm^2^ of consecutive slides. Bars represent mean±SEM. *P<0.05 and ***P<0.001 vs the respective non-damaged mucosa and ^##^ *P*<0.01 and ^###^ *P*<0.001 vs the respective mucosa in newly diagnosed patients. C) Graphs show percentage of CD86+/CD68+ cells and CD206+/CD68+ cells in newly diagnosed (n=8) and chronic patients with UC (n=12). Bars represent mean±SEM. *P<0.05 *vs* the respective non-damaged mucosa and ^&&^ *P*<0.01 and ^&&&^ *P*<0.001 *vs* the CD86/CD68 in the respective mucosa in the same graph.

Immunostaining for CD68 revealed a significant increase in the number of macrophages in the damaged mucosa of chronic patients compared with the non-damaged whereas no differences were quantified between non-damaged and damaged tissue of newly diagnosed patients (Figure 5A, B). In addition the number of macrophages in the damaged mucosa was higher in chronic than in newly diagnosed patients (Figure 5B). Analysis of CD86, a specific marker of M1 macrophages, revealed a significant increase in the damaged mucosa compared with the respective non-damaged mucosa in both, recently diagnosed and chronic patients (Figure 5B).In contrast, immunostaining of CD206, a specific M2 marker, only exhibited a significant increase in the damaged mucosa *vs* the non-damaged mucosa in chronic patients (Figure 5B). Of interest, the number of CD206 positive cells in the damaged mucosa was significantly higher in chronic patients than recently diagnosed patients. No significant differences in the proportion of CD86+/CD68+ cells nor CD206+/CD68+ cells were observed between the damaged and non-damaged mucosa of newly diagnosed patients (Figure 5C). However, a significant increase in the proportion of CD206+/CD68+ cells was detected in the damaged mucosa of chronic patients compared with the non-damaged mucosa (Figure 5C).

### Impaired enterocyte differentiation and activation of Wnt signalling pathway are observed in the damaged mucosa of chronic UC patients

**Figure 6:**
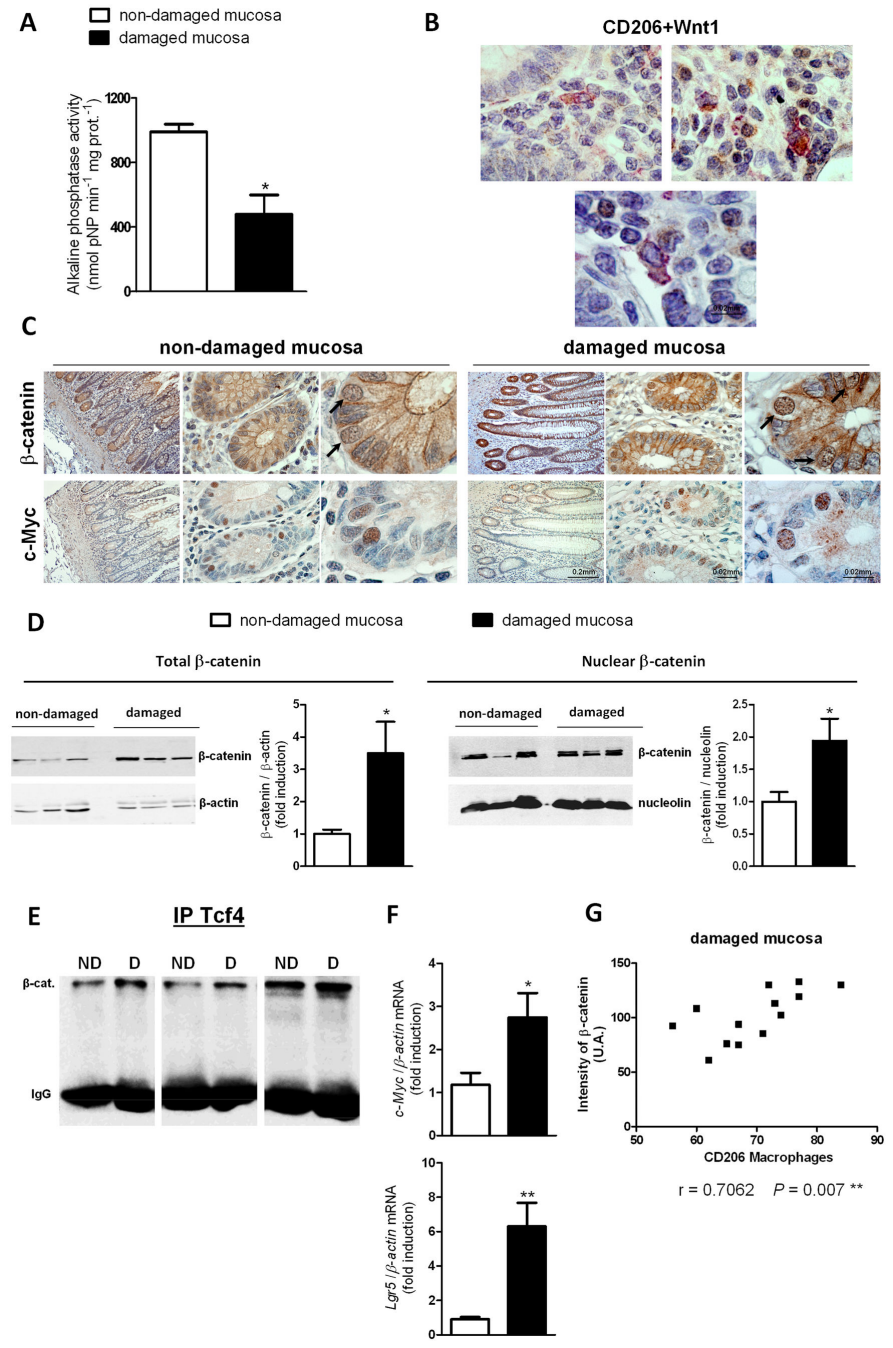
An impaired alkaline phosphatase activity and increased Wnt signalling in damaged mucosa of patients with UC. A) Graph shows alkaline phosphatase activity determined from non-damaged and damaged mucosa of chronic patients with UC. Bars represent mean±SEM. *P<0.05 vs the non-damaged mucosa (n=3). B) Three representative photographs showing co-localization of Wnt1 and CD206 in the mucosa of chronic patients with UC, magnification 160X. C) Representative photographs showing nuclear immunostaining of β-catenin and c-Myc in epithelial cells of the same crypt in consecutive slides. Magnification 10X, 60X and 160X. D) Total and nuclear protein from frozen non-damaged and damaged mucosa were extracted and expression of β-catenin was analyzed by Western blot. A representative western blot showing total and nuclear protein levels of β-catenin in the non-damaged and damaged mucosa of the same patients. Graphs show quantification of the protein expression of total (n=5) and nuclear β-catenin (n=5). E) A representative western blot showing β-catenin expression after immunoprecipitation of Tcf-4 in the non-damaged (ND) and damaged (D) mucosa of three UC patients. F) Total mRNA from frozen non-damaged and damaged mucosa was extracted. Graphs show mRNA expression of *Lgr5* and *c-Myc*. Bars represent mean±SEM. n=7. *P<0.05 and **P<0.01 vs non-damaged mucosa. G) There is a positive and significant correlation between the number of CD206 macrophages and the intensity of β-catenin immunostaining.

The analysis of the AP activity in the mucosa of chronic UC patients revealed a significant reduction in the enzymatic activity in the damaged mucosa compared with the non-damaged (Figure 6A).

To study the Wnt signalling pathway in the mucosa of chronic UC patients we first analysed the expression of Wnt1 by immunohistochemistry. Wnt1 immunostaining was detected in cells infiltrating the lamina propria and a slight staining was also observed in epithelial cells. Interestingly, double immunostaining revealed the co-localization of Wnt1 and CD206 demonstrating the expression of Wnt1 by M2 macrophages in the mucosa of UC patients (Figure 6B).

Immunohistochemistry for β-catenin showed the presence of nuclear staining in epithelial cells of the mucosa located at the base of the crypts and it decreased as cells move towards the top of the crypts (Figure 6C). The detection of nuclear immunostaining of c-Myc in epithelial cells of the same crypt in consecutive slides (Figure 6C) suggest that the Wnt signalling pathway is active in epithelial cells of the damaged mucosa of UC patients. A quantitative analysis revealed a significant increase in both total and nuclear protein levels of β-catenin (Figure 6D) and mRNA expression of *Lgr5* and *c-Myc* in damaged mucosa vs non-damaged mucosa (Figure 6F). In addition, immunoprecipitation experiments revealed an increase of β-catenin bound to Tcf-4 in damaged mucosa vs non-damaged (Figure 6E), further reinforcing that Wnt signalling pathway is stimulated in the damaged tissue. 

The specific study of Wnt signalling in the damaged mucosa highlighted a relationship between M2 macrophages and epithelial Wnt pathway. Quantification of β-catenin staining revealed a higher intensity in the damaged than in the non-damaged mucosa and results show a positive and significant correlation between the number of M2 macrophages and the intensity of epithelial β-catenin staining (Figure 6G). 

## Discussion

The present study demonstrates the selective induction of Wnt ligands by M2 macrophages and the ability of these cells to activate Wnt signalling pathways and to decrease enterocyte differentiation in adjacent epithelial cells.

The Wnt signalling pathway plays a fundamental role in intestinal epithelial proliferation and differentiation [6–8], and the induction of Wnt ligands by macrophages has recently been reported to be critical for the repair and regeneration of other tissues [13]. Our data endorse this concept and expand it by revealing a selective profile of expression of Wnt1 and Wnt3a by M2 macrophages and not by M1 cells or non-polarized macrophages. This is a consistent observation since it is observed in monocyte-derived macrophages from healthy donors and IBD patients as well as in a human cell line and suggests that a signalling pathway specifically activated by the inductor of M2 polarization, IL-4, in macrophages regulate the induction of Wnt ligands. In this line a previous study has reported activation by Il-4 of SOX2 [21], a transcription factor that has been related to the induction of Wnt1 [22].The present study demonstrates for the first time that the induction of Wnt ligands by macrophages is selectively modulated by their phenotype. 

In the intestinal mucosa, macrophages are strategically positioned to maintain communication with epithelial cells [13,23,24]. In the present study co-culture of macrophages in close proximity with epithelial cells revealed that the former modulate the latter in what appeared to be a paracrine action. Specifically M2 macrophages, and not M1 or non-polarized macrophages, significantly increased the nuclear expression of β-catenin, the central component of the canonical Wnt pathway, through Wnt1. Interestingly, the amount of β-catenin bound to Tcf-4, the main transcription factor involved in the expression of Wnt target genes, was also increased by M2 cells, as well as the mRNA expression of *Lgr5* and *c-Myc* [9,25] which strongly suggests that M2 macrophages are activating the Wnt signalling pathway in epithelial cells. It has been proposed a role for the Wnt- c-Myc pathway as an intracellular molecular switch between proliferation and differentiation [8]. Our results revealed that M2 macrophages profoundly decreased AP activity in Caco-2 cells which proposes that the spontaneous differentiation of these cells along the absorptive cell lineage [26,27] is impaired by M2 macrophages. Furthermore, this effect was mediated through the activation of canonical Wnt pathways since it was prevented by destabilization of epithelial β-catenin with XAV939 [9].The role of Wnt signalling in diminishing enterocyte differentiation is further reinforced by showing that the exogenous administration of Wnt1 to two different cell lines, Caco-2 cells and HT29 cells, significantly reduced AP activity. As a whole our results suggest that activation of β-catenin in epithelial cells repress the expression of genes associated with the onset of cell differentiation [27].

We further analysed the pathophysiological relevance of these pathways in the mucosa of UC patients. A comparative analysis performed in the damaged mucosa of newly diagnosed and chronic UC patients revealed that the number of CD68 positive cells was significantly higher in the latter than in the former. These results suggest that the number of macrophages in the mucosa of UC patients increases with the chronicity and it is not only related with the severity of damage since both chronic and newly diagnosed patients exhibited a similar histological damage. In both groups of patients, a slight increase in the number of CD86+ cells was observed in the damaged area which seems to be the consequence of the increase in the total amount of macrophages since no differences in the proportion CD86+/CD68+ cells were detected between the non-damaged and damaged mucosa. However, a significant increase in both the number of CD206+ cells and the proportion of CD206+/CD68+ macrophages was identified in the damaged mucosa of chronic patients, compared with the non-damaged tissue. This effect was not observed in the mucosa of newly diagnosed patients which suggests that the number of CD206+ cells in the damaged mucosa increases with the chronicity of the disease. These results seem to be in apparent contradiction with previous studies showing that the percentage of CD206+/CD68+ cells decreases as damage increases [28,29]. In one of those studies comparisons were performed between active and inactive Crohn's disease patients and in the other one in the same IBD patient, before and after receiving pharmacological treatment. However as far as we know the present study compares for the first time the damaged and non-damaged mucosa of the same patient and reports an increased proportion of CD206+/CD68+ cells in the injured mucosa. 

M2 macrophages are related with mucosal healing [28,29] and it has been reported that activation of the canonical Wnt pathway is an injury associated response [8,30–32] that plays an essential role in the regeneration of the mucosal damage. Of interest, our immunohistochemical studies reveal nuclear β-catenin and c-Myc immunostaining located at the base of the crypts in the damaged mucosa of UC patients which suggests that Wnt signalling is active. In addition, this pathway seems to be more activated in the damaged than in the non-damaged mucosa since the amount of nuclear β-catenin bound to Tcf-4 as well as the mRNA expression of *Lgr5* and c-Myc were significantly higher in the injured mucosa than in the non-injured mucosa. Considering that the Wnt- c-Myc pathway plays an essential role in the proliferation of both stem cells and transit-amplifying cells at the base of the crypt [5] our results suggest that this function is enhanced in the damaged mucosa of chronic UC patients in response to epithelial injury. Furthermore this pathway has been associated with diminished differentiation [6,8] and in transgenic mice that over express a Wnt inhibitor has been reported that, the inhibition of proliferation in crypt regions promotes the expression of AP at the apical domain [4]. In accordance with these observations, our results show a reduced alkaline phosphatase activity in the damaged mucosa of UC patients where Wnt signaling is stimulated. These observations joined to previous studies reporting a decrease mRNA expression of AP in human colitis [33], suggest an impaired enterocyte differentiation associated to this pathology. Both, activation of Wnt signalling and decreased enterocyte differentiation are linked to an increased number of M2 macrophages in the damaged mucosa. We found that these cells co-localized with Wnt1 in the lamina propria and in turn correlated with the intensity of β-catenin immunostaining. Although other inputs on epithelial Wnt signaling cannot be ruled out [34,35] our results propose the involvement of M2 macrophages in the activation of canonical Wnt pathways observed in the damaged mucosa of chronic UC patients. 

In summary, the present study demonstrates that M2, and not M1, macrophages activates the Wnt signalling pathway in co-cultured epithelial cells which decreases enterocyte differentiation. In the mucosa of UC patients our results reveal that M2 macrophages increase with the chronicity, act as a source of Wnt1 and they are associated with activation of Wnt signalling at the base of the crypts. In patients with prolonged and severe UC, the putative increase in the number of M2 macrophages may be involved in the development of a colorectal adenocarcinoma due to over-activation of epithelial Wnt signalling.

## Supporting Information

Table S1 **Patient characteristics.** Biopsies were obtained from patients with ulcerative colitis at the moment of diagnosis or chronic patients receiving the pharmacological treatment described at least during the last three months.
(DOC)

Figure S1 **Polarization of U937-derived macrophages towards M1 and M2 phenotypes.** U937 cells were differentiated into macrophages with PMA for 48h and treated with LPS and IFN-γ or IL-4. A) Graphs show a time course analysis of mRNA expression levels of *iNOS* and *arginase* in macrophages (n=3). (B) Graphs show a time course analysis of protein expression levels of CD86 and CD206 in macrophages (n=3). Each point represents mean±SEM. *P<0.05 and **P<0.01 vs macrophages at t=0h.
(TIF)
