# Supplementary material for: M2 Macrophages Activate WNT Signaling Pathway in Epithelial Cells: Relevance in Ulcerative Colitis
Source: PLoS One. 2013 Oct 22;8(10):e78128. doi: 10.1371/journal.pone.0078128 (PMC3805515; doi:10.1371/journal.pone.0078128)
Supplement: Table S1 — Patient characteristics. Biopsies were obtained from patients with ulcerative colitis at the moment of diagnosis or chronic patients receiving the pharmacological treatment described at least during the last three months. (DOC) [file pone.0078128.s001.doc]

**Supplementary Table 1.** Patient characteristics. Biopsies were obtained from patients with ulcerative colitis at the moment of diagnosis or chronic patients receiving the pharmacological treatment described at least during the last three months.

|  | **Newly Diagnosed** | **Chronic Patients** |
| --- | --- | --- |
| **Number of patients** | 8 | 12 |
| **Age** |  |  |
| 17-40 years | 5 | 4 |
| >40 years | 3 | 8 |
| **Gender** |  |  |
| Male | 5 | 5 |
| Female | 3 | 7 |
| **Endoscopic Score** |  |  |
| Active Colitis | 8 | 12 |
| **Location** |  |  |
| Colon | 8 | 12 |
| **Concomitant medication** |  |  |
| Corticosteroids | - | 4 |
| Mesalazine | - | 10 |
| Anti TNF | - | 3 |
