# Supplementary figures and images for: M2 Macrophages Activate WNT Signaling Pathway in Epithelial Cells: Relevance in Ulcerative Colitis
Source: PLoS One. 2013 Oct 22;8(10):e78128. doi: 10.1371/journal.pone.0078128 (PMC3805515; doi:10.1371/journal.pone.0078128)

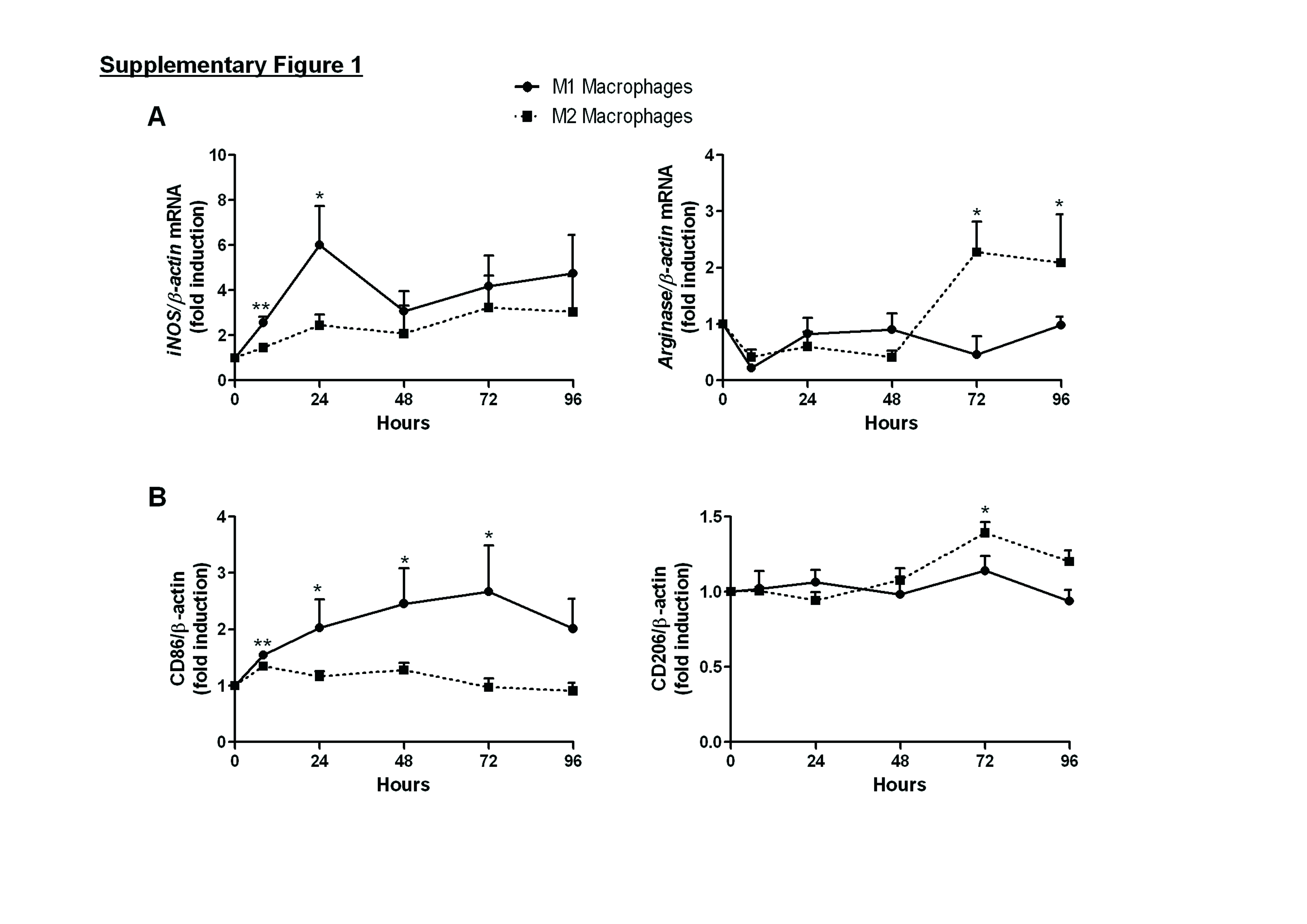

Supplement: Figure S1 — Polarization of U937-derived macrophages towards M1 and M2 phenotypes. U937 cells were differentiated into macrophages with PMA for 48h and treated with LPS and IFN-γ or IL-4. A) Graphs show a time course analysis of mRNA expression levels of iNOS and arginase in macrophages (n=3). (B) Graphs show a time course analysis of protein expression levels of CD86 and CD206 in macrophages (n=3). Each point represents mean±SEM. *P<0.05 and **P<0.01 vs macrophages at t=0h. (TIF) [file pone.0078128.s002.tif]
